# Long-read sequencing reveals atypical mitochondrial genome structure in a New Zealand marine isopod

**DOI:** 10.1098/rsos.211550

**Published:** 2022-01-12

**Authors:** William S. Pearman, Sarah J. Wells, James Dale, Olin K. Silander, Nikki E. Freed

**Affiliations:** ^1^ School of Natural and Computational Sciences, Massey University-Albany Campus, Auckland, Auckland New Zealand; ^2^ School of Environmental and Animal Sciences, Unitec Institute of Technology, Auckland, New Zealand

**Keywords:** mitochondria, heteroplasmy, palindromic, inverted repeat, isopod, asymmetry

## Abstract

Most animal mitochondrial genomes are small, circular and structurally conserved. However, recent work indicates that diverse taxa possess unusual mitochondrial genomes. In Isopoda*,* species in multiple lineages have atypical and rearranged mitochondrial genomes. However, more species of this speciose taxon need to be evaluated to understand the evolutionary origins of atypical mitochondrial genomes in this group. In this study, we report the presence of an atypical mitochondrial structure in the New Zealand endemic marine isopod, *Isocladus armatus.* Data from long- and short-read DNA sequencing suggest that *I. armatus* has two mitochondrial chromosomes. The first chromosome consists of two mitochondrial genomes that have been inverted and fused together in a circular form, and the second chromosome consists of a single mitochondrial genome in a linearized form. This atypical mitochondrial structure has been detected in other isopod lineages, and our data from an additional divergent isopod lineage (Sphaeromatidae) lends support to the hypothesis that atypical structure evolved early in the evolution of Isopoda*.* Additionally, we find that an asymmetrical site previously observed across many species within Isopoda is absent in *I. armatus*, but confirm the presence of two asymmetrical sites recently reported in two other isopod species.

## 1. Introduction

Mitochondrial genomes display a diversity of structure across Eukaryotes (reviewed in [1]), varying from multiple circular chromosomes to single linear chromosomes. However, within Bilateria, mitochondrial genomes tend to be circular in structure and contain 37 genes (13 protein-coding, two ribosomal RNAs (rRNAs) and 22 transfer RNAs (tRNAs)), with a conserved arrangement [2]. Here, we refer to this structure as ‘typical’. However, this structure and arrangement is not ubiquitous, as atypical mitochondrial arrangements have been found in some taxa. For example, booklice (Psocoptera) possess a multipartite mitochondrial genome consisting of two circular chromosomes [3]. Thrips (Thysanoptera) also possess a multipartite mitochondrial genome with massive size asymmetry (0.9 kb and 14 kb chromosomes) [4]. Tuatara, *Sphenodon punctatus*, the basally diverging lepidosaur reptile endemic to New Zealand, possesses a duplicated mitochondrial genome with a high degree of divergence between the two mitochondrial chromosomes [5].

**Figure 1.**
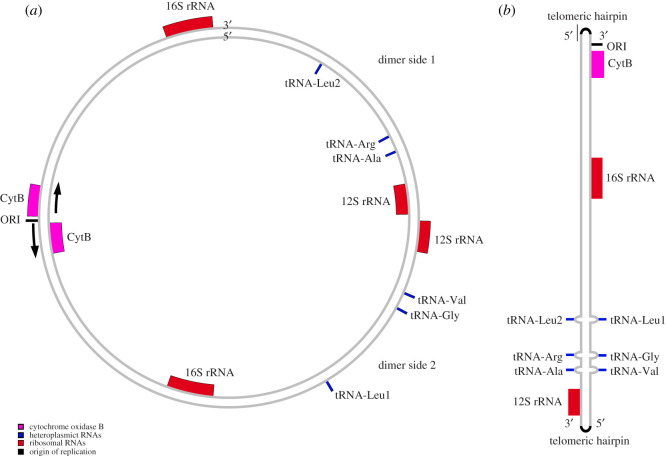
(*a*) The proposed structure of the dimer (relative sizes of the molecules not to scale). The black arrows indicate the direction of transcription and are paired with the CytB hairpin in (*b*). Putatively asymmetrical tRNA loci are shown in blue. (*b*) The proposed structure of the linear monomer, as outlined by Peccoud *et al*. [6] and Doublet *et al*. [8]. The monomer is a linearized copy of the dimer containing a telomeric hairpin. Importantly, there appear to be asymmetrical sites with mismatched bases in the linear monomer (shown in blue, and with loops at these sites), as indicated by the presence of mirrored loci coding for different tRNAs. This figure is based on the mitochondrial genome structure of *Armadillidium vulgare*, as found by Peccoud *et al*. [6].

Recent research shows that some isopods have an atypical mitochondrial genome structure (electronic supplementary material, figure S1), where each mitochondrion contains both a linear and a circular chromosome (figure 1) [6,7]. This structure is particularly common across Isopoda. The circular chromosome consists of two mitochondrial genome copies fused together in palindrome (figure 1*a*) [6]. The second, linear, chromosome (figure 1*b*) is hypothesized to be the result of linearization and self-renaturation of a single strand of the circular chromosome during replication. Self-renaturation is possible, because the circular chromosome is made of two copies that are inverted and thus self-complementary [6]. Aside from the presence of telomeric hairpins, this linear chromosome would be considered to be ‘typical’. Throughout this paper, we will refer to the circular chromosome as the ‘dimer’ and the linear chromosome as the ‘monomer’. We primarily refer to either the dimer, or the ‘unit’ which represents the fundamental repeated unit across both mitochondrial chromosomes, alongside any unique sequence between the repeats.

These different copies or structural units of the mitochondrial genome in some isopod species are not entirely identical. Peccoud *et al*. [6] have shown that there are single nucleotide differences in mirrored loci at tRNA sites, each encoding two different tRNAs. We refer to these sites as either single nucleotide polymorphisms (SNPs) (when in reference to the mitochondrial unit) or as asymmetrical sites (when in reference to the dimer).

This atypical mitochondrial genome is thought to have evolved prior to the divergence of suborders*,* such as Asellota and Oniscidea [8]. However, this hypothesis is complicated by the presence of ‘typical’ mitochondrial structures patchily dispersed in suborders such as Sphaeromatidea (family: Sphaeromatidae), Phreatoicidea (family: Amphisopodidae) and Asellota (family: Asellidae) (electronic supplementary material, figure S1) [8–13].

A second hypothesis has recently been proposed that relates the patchy distribution of mitochondrial structures across Isopoda to the unusual dominant occurrence of positive GC skews across Isopoda [14], a pattern which contrasts with other Crustacea. This second hypothesis proposes that all isopods have ancestors with atypical mitochondrial genomes in which replication occurs primarily at one copy of the duplicated control region. It has been proposed that this would lead to strand bias reversals, a process in which there are asymmetric strand-biased deaminations based on the abundance of A and C nucleotides on the lagging strand during replication [15]. Over time, this process would produce a reversed strand compositional skew and lead to a change from a negative to positive GC skew at this site. Isopod taxa that have a ‘typical’ mitochondrial genome structure, are thought to have only inherited one control region, with consequent GC skews that depend on which copy was retained. While this hypothesis necessitates regaining function of the tRNAs that are otherwise encoded for by asymmetry on the dimer, there are multiple avenues for this, such as tRNA recruitment or post-transcriptional modification [16,17].

In this study, we use long-read and short-read DNA sequencing to investigate the structure and arrangement of the mitochondrial genome of *Isocladus armatus*, a marine isopod of the Sphaeromatidae family, which is endemic to New Zealand. We show that the *I. armatus* mitochondrial genome is atypical in structure, possessing a 28 kb circular mitochondrial genome, similar to that found in other species of isopods. Because *Isocladus armatus* is highly diverged from other known lineages to possess atypical structure, it can help to resolve the evolutionary history of isopod mitochondrial genome structure. In addition, we observe two asymmetrical tRNA sites that have been observed previously [18]. However, we find no evidence of a third more widely studied asymmetry which causes a change from tRNA-Val to tRNA-Ala [18].

## 2. Methods

### 2.1. DNA extraction

Two individuals of *I. armatus* were collected from Browns Bay, Auckland in August and November of 2018. We extracted DNA from one individual for nanopore sequencing using a modified Qiagen DNEasy Blood and Tissue protocol, developed for *I. armatus* [19,20]. We extracted DNA from a second individual for Illumina sequencing using a modified Promega Wizard protocol. This protocol consisted of crushing the cephala of a specimen in a solution of chilled lysis buffer (120 µl of 0.5 M EDTA and 500 µl of the provided nuclei lysis solution), alongside 100 µl of 1 M DTT, and 30 µl of proteinase K. The crushed sample in solution was then incubated at 65°C overnight. After overnight lysis, the sample was cooled to room temperature and 10 µl of RNAse A was added, and the sample incubated at 37°C for 30 min. Following this, 250 µl of protein precipitation solution was added, and the protocol was completed according to manufacturer's instructions (page 11, Promega no. TM050).

### 2.2. Sequencing and quality control

One individual was sequenced using Oxford nanopore sequencing, and the other individual was sequenced using Illumina sequencing. For nanopore sequencing, we followed the manufacturers protocol for native barcoding of genomic DNA for the SQK-LSK109 kit (protocol version: NBE_9065_v109_revV_14Aug2019) with a R9.4 RevD flow cell. Nanopore reads were basecalled using Guppy 3.4.3, and demultiplexed and adapters removed using PoreChop (v. 0.2.4; https://github.com/rrwick/Porechop).

Illumina sequencing was carried out on an Illumina NovaSeq using 150 bp paired-end reads, with an insert size of 150 bp. Potential contaminant reads from bacteria, human, fungi or viruses were identified and discarded using Kraken2 with the maxikraken2 database (maxikraken2_1903_140GB, https://lomanlab.github.io/mockcommunity/mc_databases.html).

### 2.3. Assembly

Nanopore reads were assembled into a draft genome using Flye (v. 2.4.2; [21]) under default parameters with an estimated genome size of 1 GB. Possible mitochondrial contigs were identified by mapping all contigs to the mitochondrial genome of *Sphaeroma serratum* [10]. This identified only single contig as mitochondrial. We then mapped all nanopore reads to this contig and performed a re-assembly using only the reads that mapped to the initial mitochondrial contig. For this assembly, the default Flye settings were used with an estimated genome size of 28 kb (this size was selected based on the concordance between assembly size of the first mitochondrial contig, the size of other isopod mitochondrial genomes, and the length of the longest Nanopore reads of mitochondrial origin).

The atypical mitochondrial structure we hypothesized (shown in figure 1) precluded complete polished assemblies of this mitochondrial genome, as Illumina reads will preferentially map to the unit with fewer errors, thus Illumina reads cannot correct all errors on both units. Thus, we used Geneious 9.1 [22] to extract the linear ‘monomer’ from the assembly and manually identified the primary repeat based on a self-self dotplot of the full-length mitochondrial genome using YASS [14,23] (electronic supplementary material, figure S2). This linear monomer was manually extracted alongside any unique sequence either side of the unit in the dimer and was polished three times with Illumina reads using racon (v. 1.4.3; [24]), and BWA (v. 0.7.17; [25]) as a mapper. This contig was then visualized in IGV [26] and three putative single base pair indels (insertions/deletions) were removed based on having the depth of less than 5% of the adjacent sites.

### 2.4. Annotation

The linear monomer was then annotated using MITOS2 [27], with the Al Arab protein prediction method [28], a non-circular assembly, a final maximum overlap of 150 bp (the number of bases that genes of different types (i.e. tRNA and rRNA can overlap) and fragment overlap of 40% (the fraction of the shorter sequence that can overlap with the larger sequence). These overlap values were based on existing research indicating high levels of gene overlap within isopod mitochondrial genomes [16,29]. MITOS2 also highlights potential gene duplicates and the modification of the overlap settings may increase the likelihood of erroneously identifying gene duplication. Thus, we removed annotations for potential duplicates where there was an order of magnitude difference in quality value (analogous to a BLAST e-value, low quality values are frequently spurious (http://mitos.bioinf.uni-leipzig.de/help.py)) between annotations of potential duplicates, retaining the annotation with the highest quality factor. ARWEN was used identify any tRNAs that may have been missed with MITOS2 [30]. GC-skew was calculated to confirm the position of the origin of replication using GenSkew (https://genskew.csb.univie.ac.at/), using a step size of 100 bp and window size of 100 bp (electronic supplementary material, figure S3).

Asymmetrical sites were identified by treating them as SNPs in order to estimate the frequencies of variants at each site that differ between units of the dimer [6]. These variants were identified by mapping all Illumina reads to the 14 kb mitochondrial unit using BWA (v. 0.7.1.7; [25]), and subsequently analysed using bcftools (v. 1.10; [31]) to determine the number of reads that mapped to each variable site. Default settings were used in bcftools, except for a specification of a minimum depth of 1000 X and a maximum depth of 8000 X to call a site as a SNP.

## 3. Results

### 3.1. Assemblies

**Figure 2.**
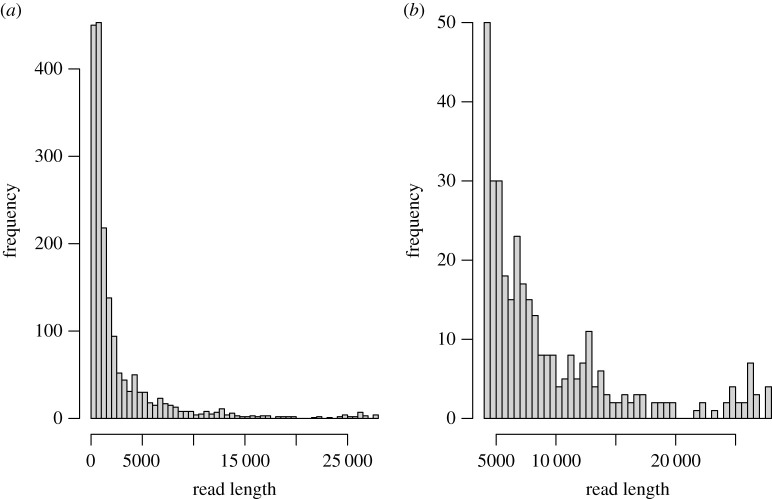
Read length distributions for all mitochondrial originating reads (*a*) and all mitochondrial originating reads greater than 5000 bp (*b*).

Nanopore sequencing of the genomic DNA from one individual was performed, and after assembly with Flye, we identified a single mitochondrial contig. One thousand eight hundred and seventeen of the nanopore reads could be mapped back to this contig (median length 1405 bp, max length 27 851 bp). The distribution of read lengths was roughly trimodal, with peaks at approximately 1400 bp, 14 000 bp and 28 000 bp (figure 2). The peak at 1400 bp is probably the result of shearing and incorporating of low molecular weight DNA in the library, while the smaller peaks observed at 14 kb and 28 kb may result from the presence of two full-length chromosomes. This hypothesis is further supported by a reduction in depth of the 28 kb dimer at the junctions between dimeric units, found in both Illumina and nanopore sequencing (electronic supplementary material, figure S4).

**Figure 3.**
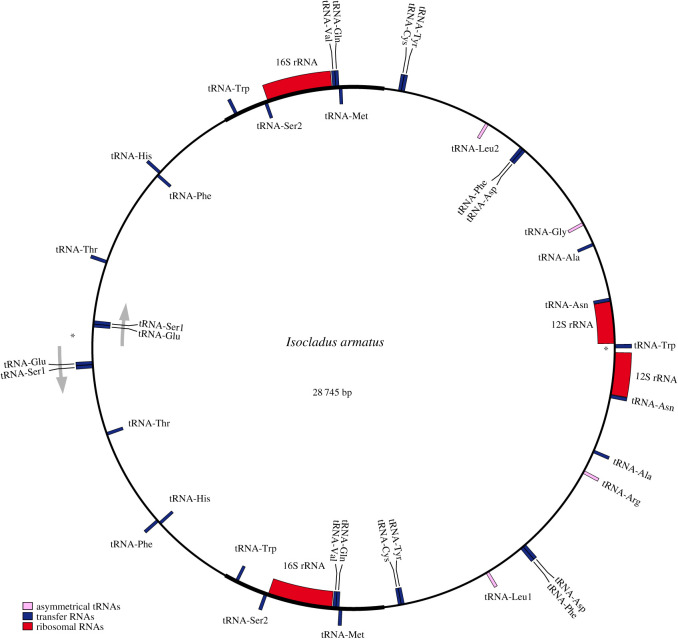
Mitochondrial genome of *Isocladus armatus*, assembled using nanopore sequencing reads. Grey arrows indicate the direction of transcription, stars indicate the positions of junctions between units, blue sites indicate transfer tRNAs, pink sites indicate tRNA loci whose function varies depending on unit of the dimer (asymmetrical sites), while red sites indicate rRNAs. Annotations were created using MITOS2, and the figure created using OGDRAW2.

Again using Flye, we performed a re-assembly using the 1817 reads that mapped back to the mitochondrial contig. This yielded a single 28 745 bp circular contig (figure 3), with a mean depth of 194 X. Annotation of this contig showed that the assembled mitochondrial genome for *I. armatus* consists of a 28.7 kb circular chromosome consisting of two inverted repeats of a ‘typical’ mitochondrial genome. The mitochondrial unit is 14 382 bp long, and the junctions between copies of the unit comprises a total of 186 bp (electronic supplementary material, figure S5). These junctions are located between each copy of the 12S gene and each copy of the tRNA-Glu gene. The junction between copies of the 12S gene is 155 bp in length, while the junction between the tRNA-Glu loci is 591 bp in length.

Each unit is composed of 13 protein-coding genes, 18 tRNAs and 2 rRNAs. Of these tRNAs, two contain variants at mirrored sites of the dimer that enable coding for a different tRNA. Finally, another tRNA locus is found within the 16S junction and is not duplicated. As a result, the complete dimer contains 13 duplicated protein-coding genes, 16 duplicated tRNAs, five unique tRNAs and two duplicated rRNAs (figure 3).

The unique component of the dimer (the ‘unit’ of the mitochondria, together with the junctions between the two copies on the dimer) was extracted and polished with racon using Illumina data, producing a linear unit 14 569 bp long (electronic supplementary material, figure S5, GenBank: OK245257). This unit represents the repeated mitochondrial unit, alongside the unique sequences found in the junctions between repeats, and has a positive GC skew (electronic supplementary material, figure S3). We selected this unit as it represents the complete unique mitochondrial genome for this species, with the exception of any single base pair heteroplasmies.

**Figure 4.**
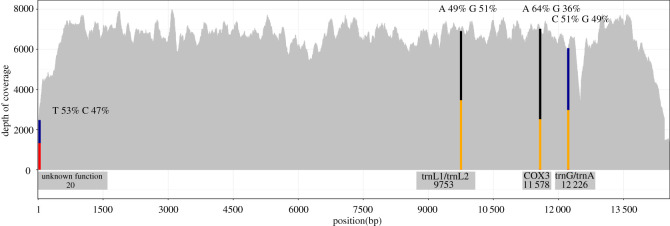
Illumina depth of the linear monomer, coloured bars indicate relative frequency of each base at the four SNP sites. Width of bars in these cases is not to scale. Captions within the figure indicate function and position within the linear monomer. Variants at SNP sites are anticipated to occur at equal abundances owing to the invariance of (or fixation) of the tRNA loci on separate units of the monomer.

The mitochondrial unit had a mean depth with Illumina reads of 6537 X (figure 4) and contained 13 protein-coding genes, 19 tRNAs and two rRNAs. Two of the tRNAs appeared asymmetrical in nature.

### 3.2. Structure of the mitogenome

We identified four single base pair mitochondrial variants (table 1) using the Illumina reads (see the electronic supplementary material, methods) (figure 4). Two of these SNPs were at tRNA loci, in the anticodon, located on either unit of the dimer, resulting in a change in the tRNA encoded at that locus. A third variant was present, with bases present with equal abundances near the junction between units of the dimer. This locus has no known function. Finally, the fourth variant was a synonymous substitution in the COX3 gene, with variant frequencies found at a 2 : 1 ratio. This latter variant was also conspicuously absent in the nanopore data (table 1). Overall, mitochondrial gene order in *I. armatus* was consistent with other isopod species.

**Table 1.** ‘SNPs' identified in the linear monomer using bcftools. (Four SNPs have been identified, three of which appear to be present in coding regions of the mitochondria.)

position	bases	function	frequency of variant- Illumina (%)	frequency of variant- nanopore (%)
20	T–C	non-coding	1315 : 1158 (53 : 47)	114^T^ : 71^C^ : 1^1^ (61 : 38 : 1)
9753	A–G	tRNA-Leu1/tRNA-Leu2	3347 : 3460 (49 : 51)	156^A^ : 113^G^ : 1^C^ : 4^T^ (57 : 41 : 0 : 2)
11 578^a^	A–G	unknown CAA-CAG	4517 : 2504 (64 : 36)	257^A^ : 4^G^ : 4^G^ : 2^C^ (96 : 2 : 2 : 0)
		Gln-Gln anticodon substitution in the COX3 gene		
12 226	C–G	tRNA-Gly/tRNA-Arg	3094 : 2963 (51 : 49)	134^C^ : 119^G^ : 6^A^ : 3^T^ (51 : 45 : 2 : 1)

^a^The presence only in Illumina sequencing data.

## 4. Discussion

Using a combination of long- and short-read DNA sequencing, we have shown that the marine isopod, *I. armatus,* exhibits an atypical mitochondrial genome structure. This atypical structure consists of a circular 28.7 kb chromosome containing two copies of a ‘typical’ mitochondria fused together as inverted repeats. This circular structure is similar in size to the mitochondrial genomes found in other isopods with atypical structure [7,18]. We identified three sites that represent differences between copies of these repeats (termed heteroplasmies). The first site is a novel single base pair substitution that occurs in a non-coding region near the junction of the repeats and appears to be unique to *I. armatus*; however, similar asymmetries have been found in the terminal regions of the duplicated regions in *Trachelipus rathkei* [18]. The other two asymmetrical sites have been previously identified in other species of isopods [6,18]. These two sites (positions 9753 and 12 226) are probably responsible for the change in tRNA function at these loci between copies of the dimer owing to a single base change in the anticodon. We also provide evidence for a putative second chromosome, which is approximately 14 kb and is, in other isopods, a linearized copy of a single mitochondrial genome (non-duplicated). We term this chromosome the linear ‘monomer’. While sequencing reads could not be assigned to the specific mitochondrial chromosome, read length distributions from nanopore sequencing suggest the existence of this linear monomer. Furthermore, reduction in depth at the junctions can be further attributed to difficulties of nanopore sequencing in sequencing the hairpin found in the monomer. Because the lengths of the junctions between units of the dimer are considerably longer than found in other species, this may introduce additional instability in the monomer—decreasing the likelihood of successfully sequencing the hairpin.

The two asymmetrical sites (tRNA-Gly-Arg and tRNA-Leu1-Leu2) we observe were recently described in three species of Oniscid isopods [18]. The presence of these sites in *I. armatus* indicates that both the atypical structure, and asymmetrical sites have been maintained over evolutionary time for hundreds of millions of years, as the most recent common ancestor between a Sphaeromatid and Oniscid isopod existed approximately 400 Ma [32]. Our data indicate that the asymmetrical sites observed in *I. armatus* have been conserved for approximately 150 Myr longer than previous estimates [8,18]. This is not particularly surprising as it is likely that this structure is maintained by purifying selection, as the loss of a tRNA would probably be lethal [6].

We identified asymmetries located on the tRNA-Arg/tRNA-Gly genes of the dimer. These sites are commonly asymmetric in species with an ‘atypical’ mitochondrial genome, therefore enabling the expression of both tRNAs [18]. However, the tRNA-Arg gene is missing in some isopod species with a ‘typical’ mitochondrial genome structure [10,11], while the tRNA-Gly gene is lacking in the Sphaeromatid isopod, *Sphaeroma terebrans* [12]. The lack of these specific tRNA loci in some isopods with ‘typical’ mitochondrial genome structures is puzzling because these lineages appear to have lost functionality of at least one tRNA locus. Despite being absent from mitochondrial gene annotations, the critical role that these tRNAs play in cellular function precludes the possibility that they are unexpressed. Instead, mechanisms such as true heteroplasmy (i.e. multiple mitochondrial haplotypes within an individual) or post-transcriptional modification may play a role in preserving the function of these genes. For example, Doublet *et al*. [8] found evidence for true heteroplasmy of the dimer (two different forms of the dimer, head to head, and head to tail) in the genus *Armadillidium*. Similar heteroplasmies may occur that could ensure functioning of putatively lost tRNAs in other species*.* Additionally, post-transcriptional modification has been proposed as an explanation for continued functionality of other tRNAs in *Ligia oceanica* [10]. This has also been directly observed in other isopods such as *Armadillidium vulgare,* where it has been found to influence the expression of the tRNA-His locus [16] In addition to asymmetries associated with tRNA function, we observed a fourth variable site that we were unable to identify as asymmetric. This SNP occurs in the COX3 gene and relates to a synonymous substitution in a tRNA-Gln anticodon. This site was only present in the Illumina sequencing data and was conspicuously absent in the nanopore data. As a result, we are unable to identify whether this site is variable between units of the dimer, as short-read Illumina data would not facilitate the determination of read orientation. The presence of this COX3 site is probably the result of intraspecific variation, as found in other species of isopods, where similar sites of asymmetry are found in massively varying levels across individuals within a species [18]. Intraspecific variation would also explain the absence of this variant within the nanopore data, as these data originate from a different individual.

Atypical mitochondrial structure, as described here, is patchily distributed across Isopoda (electronic supplementary material, figure S1), and sporadically present in various distantly related aquatic and terrestrial lineages of isopods [8,14] of both derived and ancestral origin. One hypothesis that has been proposed to explain this distribution is that atypical structure evolved early in the evolution of Isopoda, prior to the division of suborders*,* and has been subsequently lost via reversion at least three times across the order [8]. Our findings provide further support to an early origin of atypical structure, because our research confirms its presence in a marine isopod highly diverged from any other species hitherto known to possess this structure.

The mechanistic hypothesis proposed by Baillie [14] (see Introduction for discussion) that reverse GC skews found in Isopoda are the result of ancestral inheritance of a single functional control region is further supported by our data. We observe that *I. armatus* possesses a reverse GC skew to that found in many crustacea, but found frequently among isopods. Critically, because this reverse skew has now been found in another divergent isopod lineage (the Sphaeromatidae) possessing atypical structure, the hypothesis of repeated reversion to ‘typical’ mitochondrial genome structure in those species found to be possessing this structure is further supported. Alternatively, Baillie [14] suggested that many other species of isopods may have atypical structure that has been inadvertently overlooked. This is plausible because the large size of the junctions that we observe in the dimer could lead to the formation of secondary structures (i.e. telomeric hairpins) in this area which are difficult to amplify and sequence across. These secondary structures can therefore complicate the detection of atypical mitochondrial structure and lead to misidentification of the structure of the mitochondrial genome. Further studies employing long-read sequencing in other isopod species are needed to confidently determine the distribution of mitochondrial genome structures across Isopoda.

Another hypothesis, however, is that the presence of atypical structure across the order is a result of convergent evolution, that is multiple independent origins of atypical structure. This hypothesis has recently gained traction from the recent phylogenetic evidence for multiple independent terrestrial transitions within Isopoda [33,34]. This hypothesis necessitates, however, the repeated duplication of the mitochondrial unit, as well as the evolution of tRNA heteroplasmies (which appear relatively conserved across Isopoda). This explanation is therefore less parsimonious relative to the hypothesis of early origins followed by multiple reversions to the typical structure. Future long-read sequencing studies across the isopod order will be beneficial in distinguishing between these hypotheses.

## 5. Conclusion

*Isocladus armatus* possesses an atypical mitochondrial genome consisting of two chromosomes—a 28.7 kb circular dimer, and probably also a 14 kb linear monomer. The 28.7 kb dimer consists of two copies of the linear monomer fused together as inverted repeats. Three variant sites occur that differentiate the, otherwise complementary, units of the 28 kb dimer: the first of these is within a non-coding region, while the other two variants occur in two tRNA genes and cause non-synonymous changes in the tRNA anticodon between units of the dimer. The presence of this atypical structure in a Sphaeromatid isopod, alongside the differences between units of the dimer, supports the hypothesis that this structure originated early in the evolution of Isopoda, and the presence of the typical structure in some isopods may be the result of reversion to the ancestral metazoan form.

## Data accessibility

The assembled mitochondrial unit has been uploaded to GenBank under the accession: OK245257. The associated GenBank Flat File has been provided as electronic supplementary material.
